# Feasibility Study of Using Gemstone Spectral Imaging (GSI) and Adaptive Statistical Iterative Reconstruction (ASIR) for Reducing Radiation and Iodine Contrast Dose in Abdominal CT Patients with High BMI Values

**DOI:** 10.1371/journal.pone.0129201

**Published:** 2015-06-16

**Authors:** Zheng Zhu, Xin-ming Zhao, Yan-feng Zhao, Xiao-yi Wang, Chun-wu Zhou

**Affiliations:** Department of Diagnostic Radiology, Cancer Institute & Hospital, Chinese Academy of Medical Sciences (CAMS), Peking Union Medical College (PUMC), Beijing 100021, China; The University of Chicago, UNITED STATES

## Abstract

**Purpose:**

To prospectively investigate the effect of using Gemstone Spectral Imaging (GSI) and adaptive statistical iterative reconstruction (ASIR) for reducing radiation and iodine contrast dose in abdominal CT patients with high BMI values.

**Materials and Methods:**

26 patients (weight > 65kg and BMI ≥ 22) underwent abdominal CT using GSI mode with 300mgI/kg contrast material as study group (group A). Another 21 patients (weight ≤ 65kg and BMI ≥ 22) were scanned with a conventional 120 kVp tube voltage for noise index (NI) of 11 with 450mgI/kg contrast material as control group (group B). GSI images were reconstructed at 60keV with 50%ASIR and the conventional 120kVp images were reconstructed with FBP reconstruction. The CT values, standard deviation (SD), signal-noise-ratio (SNR), contrast-noise-ratio (CNR) of 26 landmarks were quantitatively measured and image quality qualitatively assessed using statistical analysis.

**Results:**

As for the quantitative analysis, the difference of CNR between groups A and B was all significant except for the mesenteric vein. The SNR in group A was higher than B except the mesenteric artery and splenic artery. As for the qualitative analysis, all images had diagnostic quality and the agreement for image quality assessment between the reviewers was substantial (kappa = 0.684). CT dose index (CTDI) values for non-enhanced, arterial phase and portal phase in group A were decreased by 49.04%, 40.51% and 40.54% compared with group B (P = 0.000), respectively. The total dose and the injection rate for the contrast material were reduced by 14.40% and 14.95% in A compared with B.

**Conclusion:**

The use of GSI and ASIR provides similar enhancement in vessels and image quality with reduced radiation dose and contrast dose, compared with the use of conventional scan protocol.

## Introduction

The concerns for the increasing potential radiation-induced malignancies [1,2,3,4,5] from CT continue to escalate since the dramatic increase of CT usage. It is said that over 30% of all CT studies were abdominal and pelvic CT examination in the United States in 2006[6]. According to risk projection models, 1.5–2.0% of all cancers in the United States may be attributable to the use of CT[2]. In particular, patients with chronic diseases such as cancer often undergo multiple CT studies in the course of follow-up, and their accumulated radiation doses for multiple studies are correspondingly increased which may cause other malignant diseases. And cancer risk from CT may no longer be theoretic because a recent study reports, for the first time, a direct increase in cancer rates related to radiation exposure from CT[7]. Therefore, radiologists should adhere to both the principle of ALARA (as low as reasonably achievable—referring to radiation dose) and the principle of AHARA (as high as reasonably achievable—referring to benefit)[8]. Iterative reconstruction algorithms such as the adaptive statistical iterative reconstruction (ASIR) have been proposed and studied to substantially reduce radiation dose without decreasing image quality in the whole body [9,10,11,12,13]. Recently, a new CT technology was introduced which combines dual-energy CT with the latest gemstone detectors for spectral imaging (GSI)[14] integrated into a 64-slice CT scanner (Discovery CT 750 High Definition; GE Healthcare, Milwaukee, WI, USA). To the best of our knowledge, the combination of GSI and ASIR in reducing radiation and contrast agent dose in abdominal pelvic CT has not been reported to date. The purpose of the present study was to prospectively investigate the effect of using GSI and ASIR for reducing radiation dose and iodine contrast dose in abdominal CT patients with high BMI values.

## Materials and Methods

### Clinical data

This prospective study was approved by the Institutional Review Board (IRB) of Cancer Institute & Hospital, Chinese Academy of Medical Sciences (CAMS) in China. Participants provided their written informed consent to this study. Between March 2014 and April 2014, 47 patients (mean age, 55±6 years; 18 men, 29 women) underwent an abdominal pelvic CT predominantly for the evaluation of abdominal cancers. Inclusion criteria were: age 18–80 years and BMI ≥ 22. Exclusion criteria were as follows: impaired renal function (eGFR < 30 ml/min), hypersensitivity to iodine contrast agents and pregnancy. Patients’ height and weight were noted and BMI (calculated as weight in kilograms divided by height in meters squared) was computed before the CT scan start. Patients were assigned to the study and control groups based on their weight and BMI combination: The study group (A) contained 26 patients (male: female = 16:10) with body weight > 65kg and BMI ≥ 22 and the control group (B) contained 21 patients (male: female = 2:19) with body weight ≤ 65kg and BMI ≥ 22. Patients in the study group were scanned with GSI mode and lower iodine load (300mgI/kg); while patients in the control group were scanned with the conventional scanning technique of 120kVp and standard iodine load of 450mgI/kg.

### CT Scans

CT scans were performed on a single-source dual-energy spectral CT scanner (Discovery CT750HD; GE Healthcare Technologies, Milwaukee, WI, USA). The scan protocol differences between groups A and B are shown in Table 1. The common scan parameters included section thickness of 5mm, layer space 5mm, FOV 35 cm, Matrix 512 ×512, detector pitch 0.984, and reconstruction thickness 1.25mm, layer space 0.8mm. For the conventional 120kVp scan, tube current was modulated to achieve a noise index (NI) of 11. All patients received a non-ionic contrast medium (Iopromide, Ultravist 300, Bayer Schering Pharma, Berlin-wedding, Germany) injected intravenously and enhanced CT was performed in the arterial and portal phase with delay times of 30 s and 65 s following the intravenous injection of contrast medium, respectively.

**Table 1:** The scan parameter and contrast agent in protocol A and B.

	Weight	KV	mAs	Contrast agent dose (ml)	Injection rate (ml/s)	NI	ASIR	Gantry rotation time
**A**	Weight > 65kg and BMI ≥ 22	GSI 60KeV	360	= 300 (mgI/kg)×weight (kg) /300 (mgI/ml)	= Contrast agent dose (ml) /30(s)	/	50%	0.5s
**B**	Weight ≤ 65kg and BMI ≥ 22	120kVp	10–700	= 450 (mgI/kg)×weight (kg) /300 (mgI/ml)	= Contrast agent dose (ml) /30(s)	11	0%	0.6s

### Qualitative and quantitative analyses

Images were reconstructed at the 60keV photon energy level with 50%ASIR for the study group and standard FBP reconstruction for the control group. Qualitative image analysis was performed by 2 independent blinded radiologists who had 25 and 10 years of abdominal CT experience. All data were randomized, rendered anonymous and were reviewed on a Picture Archiving and Communication System (PACS) diagnostic workstation (CareStream, Carestream Health, Inc, Onex, Toronto, Canada) for assessment of subjective quality. Image quality was evaluated with a scale that ranged from 1 (worst) to 5 (best) as previously reported [15]. The scoring was defined as grading point 1: poor, impaired image quality limited by excessive noise; 2: adequate, reduced image quality with either poor vessel wall definition or excessive image noise; 3: good, effect of image noise, limitation of low contrast resolution are minimal; 4: very good, good attenuation of vessel lumen and delineation of vessel walls, relative image noise is minimal; 5: excellent, clear delineation of vessel walls, limited perceived image noise. The mean value of Likert scores from the 2 observers was used for analysis.

**Fig 1:**
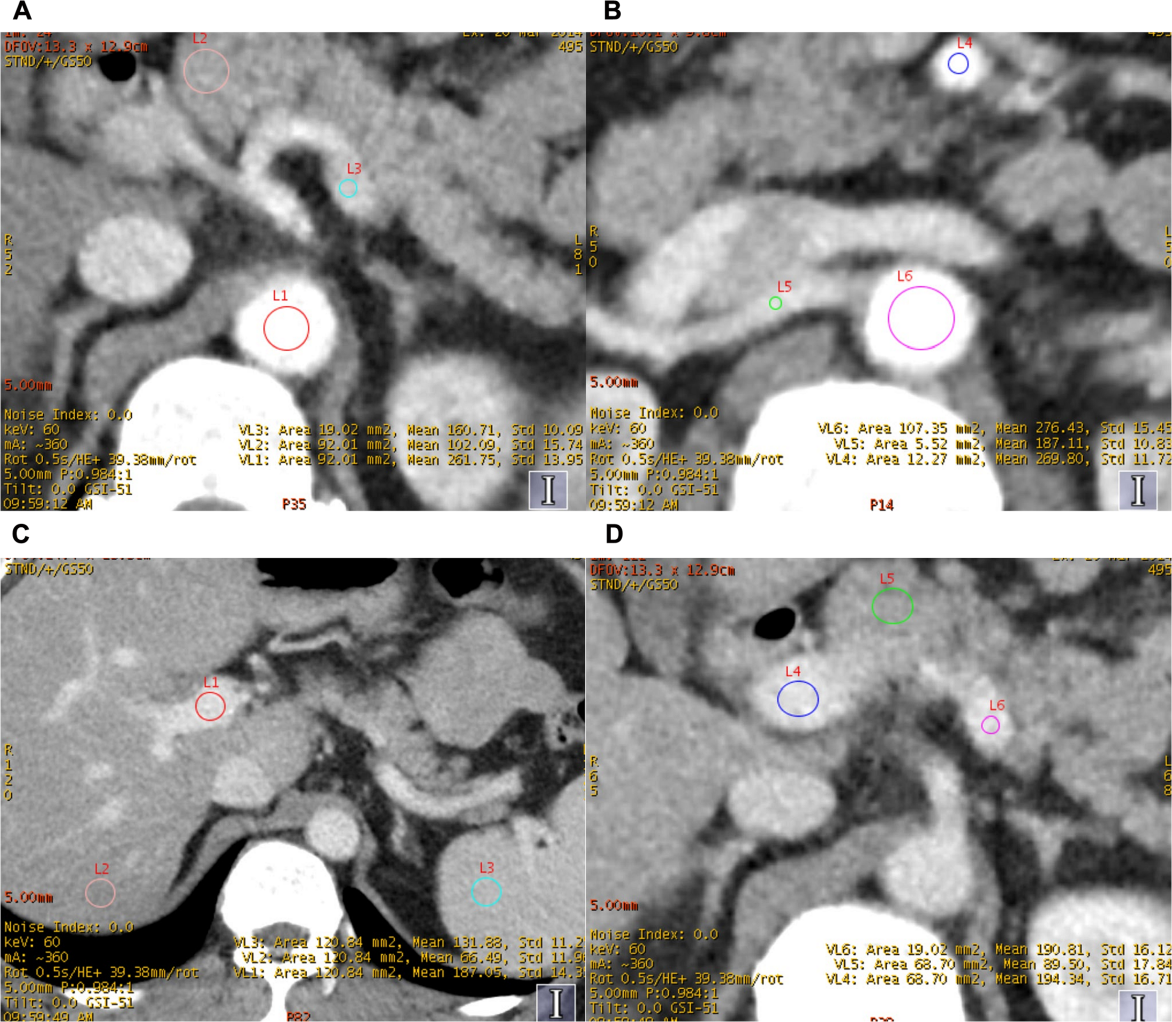
The measurement in ROI of AA1, AA2, SA, RCIA, MA, RPV, PV, SV, P-A, L-P, P-P and S-P in protocol A.

**Fig 2:**
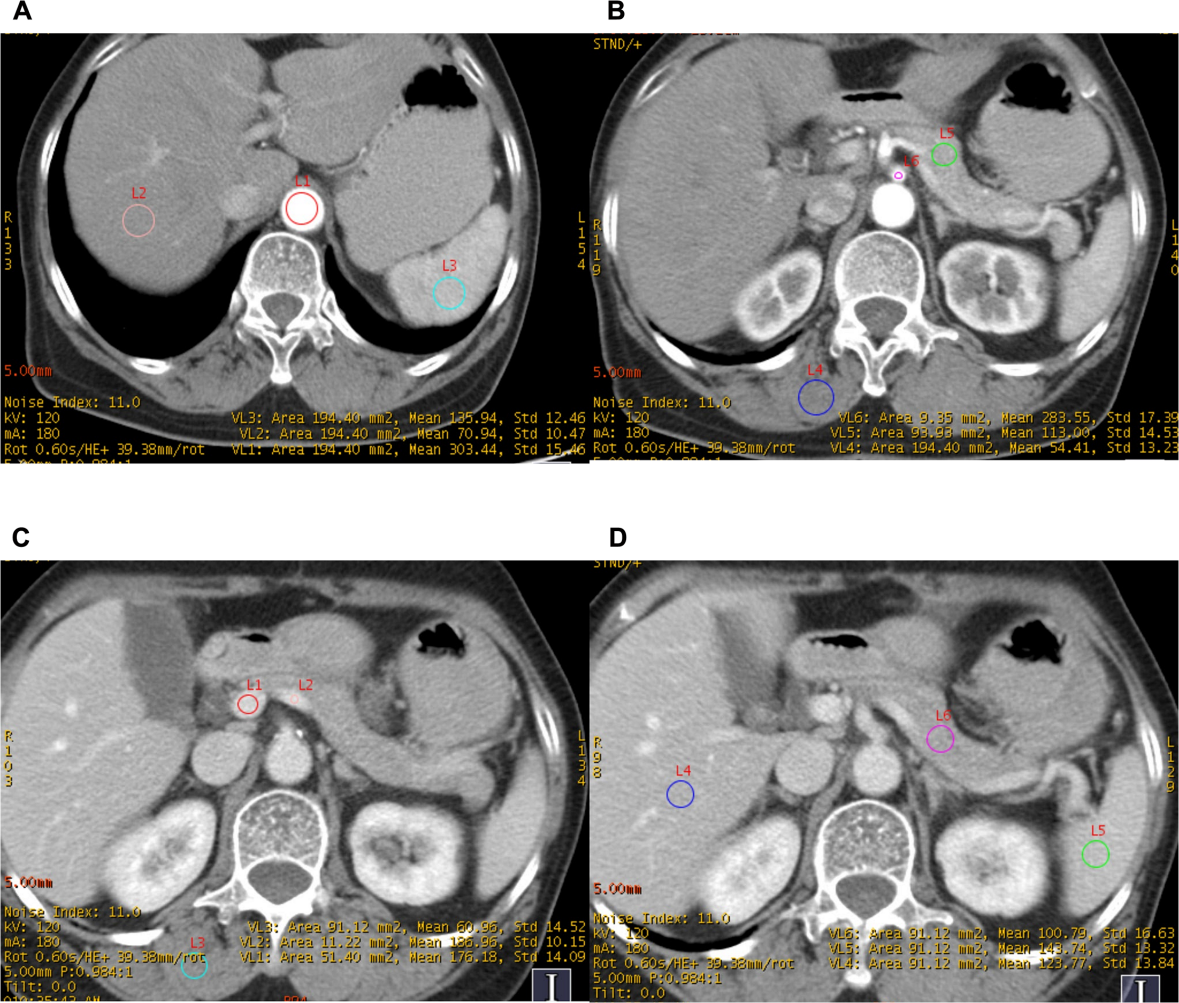
The measurement in ROI of AA1, CA, PV, SV, L-A, S-A, P-A, M-A, L-P, P-P, S-P and M-P in protocol B.

The quantitative analysis was performed by a radiologist with 10 years of imaging experience drawing regions of interest (ROI) in 26 regions, including: ⑴abdominal aorta (above celiac artery)(AA1) (Fig 1A), ⑵abdominal aorta (above aortic bifurcation)(AA2) (Fig 1B), ⑶celiac artery (CA) (Fig 2B),⑷mesenteric artery (MA)(Fig 1B), ⑸splenic artery(SA)(Fig 1A), ⑹left renal artery(LRA),⑺right renal artery(RRA) (Fig 1B),⑻left common iliac artery(LCIA), ⑼right common iliac artery(RCIA) in arterial phase; ⑽left portal vein (LPV), ⑾right portal vein (RPV) (Fig 1C), ⑿portal vein (PV)(Fig 1D), ⒀splenic vein (SV)(Fig 1D), ⒁mesenteric vein (MV) in portal phase; ⒂liver in non-contrast phase (L-N), ⒃liver in arterial phase (L-A) (Fig 2A), ⒄liver in portal phase (L-P)(Fig 1C), ⒅pancreas in non-contrast phase (L-N),⒆pancreas in arterial phase (P-A) (Fig 1A),⒇pancreas in portal phase (P-P)(Fig 1D), (21)spleen in non-contrast phase (S-N), (22)spleen in arterial phase (S-A) (Fig 2A), (23)spleen in portal phase (S-P)(Fig 2D), (24)muscle (erector spinae muscle) in non-contrast phase (M-N), (25)muscle in arterial phase (M-A)(Fig 2B) and (26)muscle in portal phase (M-P)(Fig 2C). Additionally, abdominal fat was also measured. The CT number values (in Hounsfield units, HU) and their standard deviation (SD) were measured. The ROI was placed in the region as homogeneous as possible (average of three ROI), consist 2/3 size of the vessels (ROI = 20–200 mm^2^) for measuring vessels; and avoiding the vessels or duct in the organ. CNR and SNR was calculated: CNR = (CT_target_-CT_backgroud_)/SD_background_ and SNR = CT/SD. The volumetric CT dose index (CTDIvol) and dose-length product (DLP) in dose report were also recorded [16].

Inter-observer variability was estimated by kappa statistics between the two radiologists assessing subjective image quality. The scale was following: <0.20, poor; 0.21–0.40, fair; 0.41–0.60, moderate; 0.61–0.80, substantial; and 0.81–1.00, almost perfect [17,18].

### Statistic analysis

The CT number, image noise, CNR, SNR and effective dose measurements were analyzed using the student *t*-test. When P <0.05 using version 13.0 SPSS software (SPSS, Chicago, IL USA), the difference was considered to have statistical significance.

## Results

### Clinical data

The weight, height and BMI values for patients in group A were 66–95 kg 1.55–1.80 m and 22.06–32.03 kg/m^2^, respectively. These values were all statistically higher than the corresponding values of 50–65 kg, 1.50–1.68 m and 22.03–25.81 kg/m^2^ for patients in group B (P<0.001). Ages between two protocols had no difference (P = 0.787) (Table 2).

**Table 2:** The comparison of clinical data in protocol A and B.

	Protocol	Number	Mean(Std.D)	Minimum	Maximum	*t*	P
**Age(year)**	A	26	54.70(9.60)	35	73	0.272	0.787
	B	21	53.80(13.80)	24	77		
**Height(m)**	A	26	1.69(0.078)	1.55	1.80	5.071^a^	0.000^a^
	B	21	1.60(0.049)	1.50	1.68		
**Weight(kg)**	A	26	76(7.64)	66	95	9.571	0.000^a^
	B	21	59.50(3.88)	50	65		
**BMI**	A	26	26.59(2.75)	22.06	32.03	5.753	0.000^a^
	B	21	23.23(1.05)	22.03	25.81		

BMI = body index mass (calculated as weight divided by square of height; kg/m^2^); Std.D = Std. Deviation

^a^ indicates the equal variances not assumed, and the result was *t’* test.

### Quantitative Analysis

#### CT attenuation and CNR

The enhancement in vessel is represent by CT attenuation and the potential enhancement drop due to the reduced iodine load may be compensated by the increased attenuation resulting from the lower photon energy at which the images are reconstructed. Comparison between groups A (at 60keV) and B (at 120kVp tube voltage which has average photon energy of about 70keV) (Table 3) showed that, the mean CT attenuation (Table A in S1 Table) for all 26 regions were higher in group A than B, but only values for RPV, PV and S-A were significantly different (P = 0.03, 0.04 and 0.009, respectively), representing similar or higher enhancement in the vessel in GSI group despite the decreased iodine concentration. As for the CNR, the difference between groups A and B was significant for all regions (P <0.05) except MV (P = 0.114) (Table 3, Table D in S1 Table).

**Table 3:** The comparison of HU, SD, SNR and CNR of 26 different vessels and organs in protocol A and B.

	G	HU				SD				SNR				CNR			
		Mean (Std.D)	%	*t*	P	Mean (Std.D)	%	*t*	P	Mean(Std.D)	%	*t*	P	Mean(Std.D)	%	*t*	P
**AA1**	A	303.19(54.65)	4.58	0.917	0.364	10.56(3.68)	-6.38	-0.815^a^	0.420^a^	32.43(13.53)	22.98	2.124^a^	0.041^a^	25.66(9.12)	28.04	2.346	0.023
	B	289.90(41.85)				11.28(2.26)				26.37(4.76)				20.04(6.78)			
**AA2**	A	318.23(53.16)	2.4	0.508	0.614	10.84(2.67)	-6.87	-1.126	0.266	31.09(9.48)	13.18	1.512	0.138	27.21(9.15)	24.87	2.232	0.031
	B	310.76(45.96)				11.64(2.09)				27.47(6.16)				21.79(7.01)			
**CA**	A	257.65(56.63)	4.74	0.835	0.408	16.17(6.39)	10.83	1.085^a^	0.284^a^	18.84(9.08)	4.78	0.382^a^	0.704^a^	20.77(7.81)	27.82	2.239	0.03
	B	246.00(32.88)				14.59(3.39)				17.98(6.29)				16.25(5.48)			
**MA**	A	294.15(51.26)	1.25	0.262	0.794	17.88(6.59)	23.91	1.893	0.065	18.50(6.97)	-19.88	-2.002	0.051	24.63(8.48)	23.39	2.113	0.04
	B	290.52(41.51)				14.43(5.71)				23.09(8.76)				19.96(6.18)			
**SA**	A	234.23(40.17)	9.09	1.846	0.071	14.36(4.94)	1.99	0.171	0.865	18.50(7.92)	-3.39	-0.229	0.82	18.30(6.46)	36.77	3.023	0.004
	B	214.71(30.07)				14.08(6.42)				19.15(11.70)				13.38(4.14)			
**LRA**	A	226.38(53.28)	3.84	0.498	0.621	15.25(7.06)	-5.75	-0.45	0.655	17.24(7.36)	14.93	1.17	0.248	17.55(7.58)	32.15	2.401^a^	0.021^a^
	B	218.00(62.14)				16.18(7.04)				15.00(5.32)				13.28(4.49)			
**RRA**	A	235.19(51.41)	2.92	0.391	0.698	14.75(5.45)	-22.98	-2.474	0.017	19.05(10.99)	45.87	2.34	0.024	18.58(8.30)	31.12	2.269^a^	0.029^a^
	B	228.52(65.53)				19.15(6.75)				13.06(4.52)				14.17(4.89)			
**LCIA**	A	315.62(53.42)	2.44	0.502	0.618	10.16(3.17)	-11.19	-1.487	0.144	33.89(12.30)	18.58	1.679	0.1	26.92(9.06)	24.86	2.214	0.032
	B	308.09(47.86)				11.44(2.64)				28.58(8.52)				21.56(7.08)			
**RCIA**	A	316.84(53.38)	2.89	0.597	0.554	11.19(5.67)	1.63	0.139	0.89	32.08(12.22)	8.16	0.762	0.45	27.05(9.10)	25.46	2.263	0.029
	B	307.95(47.36)				11.01(2.68)				29.66(8.89)				21.56(7.09)			
**LPV**	A	182.08(21.67)	5.77	1.687	0.099	11.32(2.91)	0.27	0.049	0.961	17.05(4.53)	7.98	1.027	0.31	12.09(3.34)	18.76	2.092	0.042
	B	172.14(17.89)				11.29(2.02)				15.79(3.69)				10.18(2.81)			
**RPV**	A	181.69(20.59)	8.15	2.292	0.027	10.58(3.04)	-14.4	-2.215	0.032	18.66(6.24)	32.25	3.006	0.004	12.01(3.02)	23.31	2.649	0.011
	B	168.00(20.07)				12.36(2.34)				14.11(3.34)				9.74(2.79)			
**PV**	A	185.08(19.79)	7.52	2.126	0.039	11.70(1.83)	-2.5	-0.631	0.531	16.14(2.67)	11.54	2.29	0.027	12.40(3.22)	21.33	2.285	0.027
	B	172.14(21.87)				12.00(1.34)				14.47(2.23)				10.22(3.32)			
**SV**	A	181.08(21.13)	5.69	1.566	0.124	10.60(2.99)	-13.26	-1.818	0.076	18.57(6.45)	23.97	2.079	0.043	11.96(3.27)	18.77	2.033	0.048
	B	171.33(21.29)				12.22(3.08)				14.98(5.09)				10.07(3.01)			
**MV**	A	175.69(20.92)	3.84	1.102	0.276	11.45(3.21)	1.42	0.19	0.85	16.38(4.42)	3.34	0.406	0.686	11.34(3.06)	14.43	1.613	0.114
	B	169.19(19.05)				11.29(2.54)				15.85(4.52)				9.91(3.00)			
**L-N**	A	58.23(8.99)	0.64	0.146	0.884	9.49(1.55)	-15.27	-3.167	0.003	6.35(1.67)	19.14	2.494^a^	0.016^a^				
	B	57.86(8.34)				11.20(2.15)				5.33(1.14)							
**L-A**	A	70.15(12.46)	1.04	0.205	0.838	9.71(1.75)	-17.92	-4.671	0	7.53(2.39)	28.28	3.314^a^	0.002^a^				
	B	69.43(11.48)				11.83(1.24)				5.87(0.84)							
**L-P**	A	116.58(15.00)	5.39	1.335	0.189	10.16(1.95)	-17.67	-3.908	0	11.95(2.91)	30.32	3.66	0.001				
	B	110.62(15.47)				12.34(1.83)				9.17(2.11)							
**P-N**	A	52.73(4.31)	3.21	1,368	0.178	9.68(1.84)	-3.97	-0.734	0.467	5.62(1.10)	6.64	1.03	0.308				
	B	51.09(3.75)				10.08(1.86)				5.27(1.26)							
**P-A**	A	129.00(13.85)	7.16	1.963	0.056	14.89(4.61)	-9.705	-1.214	0.231	9.49(3.08)	21.2	2.025	0.049				
	B	120.38(16.25)				16.49(4.39)				7.83(2.43)							
**P-P**	A	130.00(10.98)	2.98	1.058	0.296	10.18(2.58)	-7.71	-1.364	0.179	13.45(3.11)	15.85	2.520^a^	0.016^a^				
	B	126.24(13.41)				11.03(1.37)				11.61(1.84)							
**S-N**	A	43.86(6.81)	2.79	0.602	0.55	10.33(2.64)	-4.53	-0.75	0.457	4.41(0.89)	9.7	1.453	0.153				
	B	42.67(7.93)				10.82(1.56)				4.02(0.93)							
**S-A**	A	108.69(11.81)	8.69	2.736	0.009	11.26(2.39)	-3.01	-0.524	0.603	9.34(3.01)	19.28	1.858	0.07				
	B	100.00(9.45)				11.61(2.16)				7.83(2.43)							
**S-P**	A	99.73(10.77)	1.67	0.525	0.602	11.32(3.11)	-8.93	-1.391	0.171	9.52(3.12)	17.82	2.088^a^	0.044^a^				
	B	98.09(10.42)				12.43(2.16)				8.08(1.44)							
**M-N**	A	55.42(6.35)	3.92	1.262	0.213	10.23(2.23)	-10.49	-1.843	0.072	5.71(1.58)	19.21	2.541^a^	0.015^a^				
	B	53.33(4.61)				11.43(2.19)				4.79(0.83)							
**M-A**	A	62.69(6.54)	3.19	1.057	0.296	9.80(1.66)	-18.33	-3.893	0	6.59(1.36)	26.25	3.828	0				
	B	60.75(5.88)				12.00(2.21)				5.22(1.03)							
**M-P**	A	69.54(5.67)	0.71	0.257	0.798	9.62(1.73)	-7.85	-1.703	0.095	7.48(1.64)	11.48	2.028^a^	0.049^a^				
	B	69.05(7.43)				10.44(1.51)				6.71(0.95)							

Note.—The mean CT numbers are expressed in Hounsfield units. Numbers in parentheses are standard deviations.

AA1 = abdominal aorta (above celiac artery); AA2 = abdominal aorta (above the aortic bifurcation); CA = celiac artery; MA = mesenteric artery; SA = splenic artery; LRA = left renal artery; RRA = right renal artery; LCIA = left common iliac artery; RCIA = right common iliac artery; LPV = left portal vein; RPV = right portal vein; PV = portal vein; SV = splenic vein; MV = mesenteric vein; L-N = liver in non-contrast phase; L-A = liver in arterial phase; L-P = liver in portal phase; P-N = pancreas in non-contrast phase; P-A = pancreas in arterial phase; P-P = pancreas in portal phase; S-N = spleen in non-contrast phase; S-A = spleen in arterial phase; S-P = spleen in portal phase; M-N = muscle in non-contrast phase; M-A = muscle in arterial phase; M-P = muscle in portal phase.

^a^ Value shows a statistically significant difference with a two-tailed P value of less than 0.05, compared with the value of protocols A and B combined. It means the equal variances not assumed, and the result was *t’* test.

G = group; HU = hounsfield unit; Std.D = Std. Deviation; SD = Std. Deviation of HU; SNR = signal to noise ratio; CNR = contrast to noise ratio; % = (A-B)/B×100%.

#### SNR and SD

The image noise is reflected by the SNR and SD. The higher the SNR, the better the image was. Table 3 and Table C in S1 Table showed that SNR values were higher in group A than B except for MA and SA. The difference of SNR of AA1, RRA, RPV, PV, SV, L-N, L-A, L-P, S-A, S-P, P-P, M-N, M-A and M-P was statistically significant in group A compared with B (P <0.05), other regions had no significant difference. The image noise, reflected by SD, was higher for images in group B (Table B in S1 Table). Although the contrast material dose was reduced by 14.40% in group A (Table 4, Table in S2 Table), the CT value were higher except for CA, MA, SA, RCIA, LPV and MV. The difference between groups A and B was statistically significant for the RRA, RPV, L-N, L-A, L-P and M-A (P <0.05), other tissue had no significant difference.

**Table 4:** The comparison of contrast agent dose and radiation dose (DLP) in protocol A and B.

	Protocol	N	Mean	Std. D	%	*t* ^*'*^	P value
**Total dose of injection(ml)**	A	26	76.92	8.68	-14.4	-6.373	0
	B	21	89.86	5.07			
**Velocity of contrast agent(ml/s)**	A	26	2.56	0.31	-14.95	-6.456	0
	B	21	3.01	0.17			
**CTDI** _**n**_ **(mGy)**	A	26	10.34^b^	-	-49.04	-11.929	0
	B	21	20.29	3.82			
**CTDI** _**a**_ **(mGy)**	A	26	10.34	-	-40.51	-9.68	0
	B	21	17.38	3.34			
**CTDI** _**p**_ **(mGy)**	A	26	10.34	-	-40.54	-9.692	0
	B	21	17.39	3.33			
**DLP** _**n**_ **(mGy.cm)**	A	26	505.55	62.31	-47.51	-10.246	0
	B	21	963.12	196.84			
**DLP** _**a**_ **(mGy.cm)**	A	26	519.43	26.80	-36.86	-8.038	0
	B	21	822.67	171.18			
**DLP** _**p**_ **(mGy.cm)**	A	26	519.43	26.80	-36.83	-8.04	0
	B	21	822.26	170.92			
**DLP** _**t**_ **(mGy.cm)**	A	26	1552.53	77.23	-40.68	-8.987	0
	B	21	2617.11	538.41			

^b^ indicate group A = fixed dose, CTDI = 10.34

CTDI_vol_ = CT dose index volume; DLP = dose length product

_n_ = non-enhanced phase

_a_ = arterial phase

_p_ = portal phase

_t_ = total.

N = Number; Std.D **=** Std. Deviation. % = (A-B)/B×100%.

### Qualitative Analysis

All image quality scores were ≥ 4 (very good) with respect to the overall image quality and enhancement of the abdominal organs. There was substantial inter-observer agreement with respect to image quality (κappa = 0.684). Using the scores of the radiologist with 25-years work experience as standard, 26/26 in group A scored 4; 11/21 in group B scored 5 and 10/21 scored 4. Scores for group B were higher than A with statistical significance (χ^2^ = 17.780, P = 0.000).

### Radiation Dose and Contrast Agent Dose

According to the manufacturer’s data, the volumetric CT dose index (CTDIvol) in the non-contrasted, arterial and portal phase for group A was decreased by 49.04%, 40.51% and 40.54% compared with B (P = 0.000) (Table 4). Similarly, the dose length product (DLP) were also decreased by 47.51%, 36.86%, 36.83%, and the average dose reduction was 40.68% for group A (P = 0.000) (Table 4, Table C, D in S2 Table). The total dose and the injection rate for the contrast material in group A had a reduction of 14.40% and 14.95%, respectively, compared with group B (Table 4, Table A, B in S2 Table).

## Discussion

In this study we evaluated the feasibility of combining dual energy spectral CT imaging with ASIR to reduce both the radiation dose and contrast medium dose. Our results demonstrated that compared with the conventional imaging and reconstruction technique the combination of spectral CT and ASIR reduced the radiation dose and contrast medium dose by 41% and 14%, respectively. The low energy monochromatic images effectively compensated the lower contrast medium dose in the study group. ASIR, which provides the real-time reconstruction and improved the image quality [13,19,20], has now largely been implemented into routine clinical practice. Our study showed that ASIR reduced the abdominal image noise in the lower energy images to yield higher contrast-noise-ratio value for patients in the study group with spectral CT imaging. All images in both the study group and control group had diagnostic image quality, even though the control group had higher image quality scores probably due to the fact that the overall BMI value in the control group was smaller.

Lower objective noise levels using ASIR resulted in increased SNR. In addition, by adapting ASIR into our protocols, we were able to reduce the radiation dose and contrast material dose without compromising objective image quality. The higher the SNR, the better the image was. ASIR overcame some of the disadvantages that inversely affect SNR for abdominal CT scans in the study group, such as higher BMI, lower contrast material dose and lower radiation dose, the resulting SNR for the study group was better or not worse than the control group, which had smaller BMI and with regular contrast dose and radiation dose.

Nakayama’s study showed that by decreasing the tube voltage, the amount of contrast material can be reduced without image quality degradation[21]. However, images obtained using lower tube tend to be noisier, mainly because of the higher absorption of low-energy photons by the patient, which requires an upward adjustment of the tube current to avoid any deprivation in image quality. The use of low keV images in spectral CT exhibits similar behavior. Low keV setting provides high conspicuity of contrast materials at CT but results in higher image noise, particularly in larger patients[22]. In our study, for heavy patients, the image noise was increased and image quality was reduced due to the use of a fixed spectral CT scan protocol. But the mean image quality score was still greater than 4; thus, clinical confidence was maintained.

There are limitations to our study. The small sample size requires confirmation in larger series. Another limitation is that the dose reduction numbers are apply only to the task in this study and the conclusion may only apply to the equipment and algorithm used in the study. The third limitation is that the group without ASIR and GSI images appeared smoothed and the radiologists were more accustomed to. It is possible; therefore, that these differences in image appearance may have allowed the two reviewers to distinguish between images with or without ASIR and GSI despite the randomization of image sets. And maybe this is the reason why the result of subjective and objective evaluation of image was not the same. Moreover, the patients enrolled in our study were all Asian person, which usually have smaller BMI, and that is the reason why our study choose BMI 22 as a dividing line, in future, we plan to include patients with larger BMI, which may be more representative and further determine whether our results also apply to heavier patients.

In conclusion, the present study found that the use of GSI and ASIR provides similar enhancement in vessels and image quality with reduced radiation dose and contrast dose, compared with the use of conventional scan protocol. Although the dose reduction numbers obtained in this study apply only to the specific patient group, equipment and algorithm used, the principle could be applied to other patient populations, especially for those patients who may need to undergo multiple CT examinations and are at increased risk for developing cancer from medical radiation exposure.

## Supporting Information

S1 Table the histogram of HU, SD, SNR and CNR of 26 different vessels and organs in protocol A and B. (A) Comparison between groups A and B showed that the mean CT attenuation for all 26 regions were higher in group A than B. (B) SD in protocol A was lower than B except for CA, MA, RCIA, LPV and MV. (C) SNR values were higher in group A than B except for MA and SA. (D) CNR in groups A were all higher than B.
(DOC)

S2 Table the error bar of contrast agent dose, velocity of contrast agent and radiation dose (DLP) for different phase and total in protocol A and B. The total dose (A) and the injection rate (B) for the contrast material in group A had a reduction compared with group B. The DLP for non-enhanced phase, arterial phase, portal phase (C) and totoal phase (D) were also decreased for group A.
(DOC)
