# Supplementary material for: Feasibility Study of Using Gemstone Spectral Imaging (GSI) and Adaptive Statistical Iterative Reconstruction (ASIR) for Reducing Radiation and Iodine Contrast Dose in Abdominal CT Patients with High BMI Values
Source: PLoS One. 2015 Jun 16;10(6):e0129201. doi: 10.1371/journal.pone.0129201 (PMC4469609; doi:10.1371/journal.pone.0129201)
Supplement: S1 Table — (A) Comparison between groups A and B showed that the mean CT attenuation for all 26 regions were higher in group A than B. (B) SD in protocol A was lower than B except for CA, MA, RCIA, LPV and MV. (C) SNR values were higher in group A than B except for MA and SA. (D) CNR in groups A were all higher than B. (DOC) [file pone.0129201.s001.doc]

**S1 Table. the histogram of HU, SD, SNR and CNR of 26 different vessels and organs in protocol A and B**

**A B**

**C D**
