# Supplementary material for: Feasibility Study of Using Gemstone Spectral Imaging (GSI) and Adaptive Statistical Iterative Reconstruction (ASIR) for Reducing Radiation and Iodine Contrast Dose in Abdominal CT Patients with High BMI Values
Source: PLoS One. 2015 Jun 16;10(6):e0129201. doi: 10.1371/journal.pone.0129201 (PMC4469609; doi:10.1371/journal.pone.0129201)
Supplement: S2 Table — The total dose (A) and the injection rate (B) for the contrast material in group A had a reduction compared with group B. The DLP for non-enhanced phase, arterial phase, portal phase (C) and totoal phase (D) were also decreased for group A. (DOC) [file pone.0129201.s002.doc]

**S2 Table. the error bar of contrast agent dose, velocity of contrast agent and radiation dose (DLP) for different phase and total in protocol A and B**

**A B**

**C D**
